# The German fibromyalgia consumer reports – a cross-sectional survey

**DOI:** 10.1186/1471-2474-13-74

**Published:** 2012-05-18

**Authors:** Winfried Häuser, Eva Jung, Brigitte Erbslöh-Möller, Mechthild Gesmann, Hedi Kühn-Becker, Franz Petermann, Jost Langhorst, Reinhard Thoma, Thomas Weiss, Frederick Wolfe, Andreas Winkelmann

**Affiliations:** 1 Department Internal Medicine I, Klinikum Saarbrücken, Saarbrücken, D-66119, Germany; 2 Department of Psychosomatic medicine and psychotherapy, Technische Universität München, München, D-81675, Germany; 3 Rheumatology Office, Neunkirchen, D-66538, Germany; 4 Psychosomatic medicine and pain therapy office, Herford, D- 32049, Germany; 5 Psychosomatic medicine and pain therapy office, Zweibrücken, D-66432, Germany; 6 Centre of clinical psychology and rehabilitation, Universität Bremen, Bremen, D- 28359, Germany; 7 Department Internal Medicine V (Integrative Medicine), Kliniken Essen-Mitte, Universität Essen-Duisburg, Essen, D-45726, Germany; 8 Algesiologikum München, München, 80799, Germany; 9 Day clinic Mannheim Dr. Weiss, Mannheim, D-68161, Germany; 10 National Data Bank for Rheumatic Diseases, Wichita, Kansas and University of Kansas School of Medicine, Wichita, KS, USA; 11 Department of Physical Medicine and Rehabilitation, Klinikum der, Universität München, München, D-80336, Germany

**Keywords:** Fibromyalgia syndrome, Consumer reports, Drugs, Non-pharmacological therapies

## Abstract

**Background:**

Consumer surveys provide information on effectiveness and side effects of medical interventions in routine clinical care. A report of fibromyalgia syndrome (FMS) consumers has not been carried out in Europe.

**Methods:**

The study was carried out from November 2010 to April 2011. Participants diagnosed with FMS rated the effectiveness and side effects of pharmacological and non-pharmacological FMS interventions on a 0 to 10 scale, with 10 being most efficacious (harmful). The questionnaire was distributed by the German League for people with Arthritis and Rheumatism and the German Fibromyalgia Association to their members and to all consecutive FMS patients of nine clinical centers of different levels of care.

**Results:**

1661 questionnaires (95% women, mean age 54 years, mean duration since FMS diagnosis 6.8 years) were analysed. The most frequently used therapies were self-management strategies, prescription pain medication and aerobic exercise. The highest average effectiveness was attributed to whole body and local warmth therapies, thermal bathes, FMS education and resting. The highest average side effects were attributed to strong opioids, local cold therapy, gamma-amino-butyric acid analogues (pregabalin and gabapentin), tramadol and opioid transdermal systems.

**Conclusion:**

The German fibromyalgia consumer reports highlight the importance of non-pharmcological therapies in the long-term management of FMS, and challenges the strong recommendations for drug therapies given by FMS-guidelines.

## Background

The key symptoms of fibromyalgia syndrome (FMS) are chronic widespread pain, unrefreshed sleep, cognitive dysfunction and fatigue [1,2]. Patients often report high disability levels and poor quality of life along with extensive use of medical care [3,4]. The prevalence of FMS was 2.9-3.8% in the general population of five European countries [5,6]. FMS can be diagnosed in clinical care by the American College of Rheumatology 2010 preliminary diagnostic criteria [1] and in surveys by the fibromyalgia survey diagnostic criteria [7].

The definitive etiology and pathophysiology of FMS are unknown [8]. A great variety of pharmacological and non-pharmacological therapies are offered to and used by patients to relieve symptoms [9]. Evidence-based guidelines aim to guide patients and health care providers in the choice of treatment options [10-12]. These guidelines rely on randomised controlled trials of pharmacological and non-pharmacological therapies. However, the external validity of RCTs in FMS is limited: Most studies excluded patients with inflammatory rheumatic diseases and severe mental disorders and were conducted in research and university centers [13]. Consumer reports, in which patients assess the effectiveness and side effects of interventions, can supplement the results of RCTs, because participants are not excluded because of comorbidities [13]. The US National Fibromyalgia Association (NFA) conducted an internet survey in which 2596 participants responded about the effectiveness of FMS-treatments in 2005. Side effects were not assessed [14].

Given the limited external validity of RCTs in FMS, we conducted the first European FMS consumer reports on the effectiveness and side effects of FMS-therapies in routine clinical care.

## Methods

### Recruitment

Participants of the study were recruited by the two largest German FMS-self help organisations and nine clinical institutions. The specialties of the clinical institutions were pain medicine and psychotherapy (N = 3), rheumatology (N = 2), complementary and alternative medicine (N = 2), physical therapy (N = 1) and pain therapy (N = 1). The settings were outpatient (N = 6), inpatient (N = 2) and day clinic (N = 1). The levels of care were secondary (N = 6) and tertiary care (N = 2) and rehabilitation (N = 1).

### Inclusion- and exclusion criteria

Members of the self-help organisations had to report that the diagnosis of FMS had been established by a physician. Because there is no gold standard for the *clinical* diagnosis of FMS [15], the physicians of the participating study centers were free in their choice of FMS-diagnostic criteria. Patients who were not able to read German and patients with a FMS-diagnosis of <1 month’s duration were excluded. There were no other exclusion criteria.

### Procedure

#### Clinical institutions

From November 1, 2010 to April 30, 2011 all consecutive patients with an established diagnosis of FMS at the participating study centres were asked by the physicians of these centres to take part in the study. The questionnaires were handed out by the physicians of the centres with a standardized letter explaining the focus of the study. The questionnaires were returned by the patients in a closed and anonymous envelope and kept away from the charts. At the end of the study the questionnaires were sent to the coordinating study centre.

#### Self-help organisations

The package of questionnaires was sent by the central office of the German League for people with Arthritis and Rheumatism to their regional offices with the request that the leaders of the local self-help groups distribute the questionnaires to their members during group meetings. The German Fibromyalgia Association included the package in the 4/2010 issue of its member journal „Optimist,“which was sent out by post to all members. Patients returned the questionnaires by post at their own cost to the central office. In addition, the questionnaires were available on the homepages of both self-help organisations. After downloading and completing the completed questionnaires, they were sent by mail, fax or email to the central offices. Employees of both central offices removed personal identifying information and sent the questionnaires to the coordinating study centre.

The participants (investigators and patients) of the study did not receive any reimbursement or compensation for participation.

### Survey questionnaires

Demographic data and medical data were assessed by a questionnaire used in a previous multicenter German FMS-study [16].

The questionnaire “Benefits and harms of FMS-therapies” was delevoped by the heads of the clinical centers taking part in the study and by the directors of the two FMS self-help organisations. Similar to the NFA – questionnaire [14], survey participants were asked to "indicate whether you currently use any of the following interventions for FMS and if so, whether each helps to relieve your FMS symptoms". Moreover, the patients were asked if they had used these interventions in the past. Respondents rated the effectiveness (relief of symptoms) and harms (side effects) of each intervention used in the present or past on a 0 to 10 scale, with 10 being most effective (harmful). The interventions were listed in different sections: Self-management strategies (exercise, resting, physical modalities), psychological therapies, drugs and complementary/alternative medicine (CAM) (a copy of the questionaire is available on request). Face validity and understandability of the questionnaire were qualitatively validated by four clinicians (rheumatology, pain medicine, psychosomatic medicine) and 10 patients (integrative medicine, pain medicine) not involved in the study. Eight questioniare items were modified according to the validation reports.

The 4-item Patient Health Questionnaire-4 (PHQ-4) is an ultra-brief self-report questionnaire that consists of a 2-item depression scale (PHQ-2) and a 2-item anxiety scale (GAD-2). A score of ≥ 3 on the depression subscale represents a reasonable cut-point for identifying potential cases of major depression or other depressive disorders; a score of ≥3 on the anxiety subscale represents a reasonable cut-point for generalized anxiety, panic, social anxiety, and posttraumatic stress disorders [17]. We used the validated German version of the PHQ 4 [18].

### Statistical analysis

The data were entered by four pairs of study assistants into a preconstructed data sheet. The entering of data was randomly checked by two authors, and further checked for plausibility during descriptive data analysis. Missing values were not replaced. Descriptive statistics were performed by Winstat for Excel (R. Fitch Software, Bad Krozingen, Germany, 2010).

### Ethics

Study investigators respected the requirements of data protection and medical professional secrecy. The study was approved by the ethical committee of the Ludwig Maximilian Universität München.

## Results

### Study participants

There were no data available about how many patients contacted by the self-help organisation declined to participate in the study. The German League for people with Arthritis and Rheumatism estimated that 10,000 of their members were FMS patients. The German Fibromyalgia Association indicated that 4,000 members have FMS.

123 patients of the clinical samples did not meet the primary inclusion criteria and 40 of the contacted patients refused to take part in the study. 1,661 questionnaires were analysed. The total study sample was composed mainly of middle-aged women with long durations of CWP and time from FMS diagnosis (see Table 1).

**Table 1:** Demographic and clinical characteristics of the study participants (N = 1661)

	N (%) *	**Mean (Standard deviation; range)**
**Sex**		
Female	1573 (95.2)	
Male	80 (4.8)	
**Age**	1650	54.3 (9,8; 19,86)
**Family situation**		
Living with a partner	1249 (75.8)	
Living alone	377 (22.9)	
Living in the family	21 (1.3)	
**School degree**		
None	26 (1.6)	
Primary	559 (34.0)	
Secondary school	675 (41.0)	
High school	132 (8.0)	
University	252 (15.3)	
**Current professional situation**		
Student	10 (0.6)	
Working	532 (32.3)	
Sick leave	131 (8.0)	
Applying for disability pension	150 (9.1)	
Without job	21 (1.3)	
House wife	180 (11.0)	
Pensioneer	620 (37.7)	
**Years since chronic widespread pain**	1634	16.6 (11.1; 0.25-61)
**Years since diagnosis of fibromyalgia syndrome**	1601	6.8 (5.5; 0.1-41)
**Member FMS-self help organisation**	1017 (61.2)	

* Note: The discrepancies between the number of persons included in the study and the number of persons in the following rows are due to missing items.

1411 (85.5%) participants met the fibromyalgia survey diagnostic criteria [7]. 276/1351 (20.4%) participants reported a diagnosis of an inflammatory rheumatic disease. 881/1633 (54.6%) participants scored > = 3 on the PHQ 4 depression scale and 889/1633 (54.4%) scored > = 3 on the PHQ 4 anxiety scale.

### Most frequently used interventions

The most frequently used types of current interventions were self-management strategies, prescription pain medication and aerobic exercise (see Table 2).

**Table 2:** Currently used types of management strategies

**Type of therapy**	N *	% *
Self-management, activity-based (e.g. promenading, distraction)	1542/1618	95.3
Self-management, rest-based (e.g. lying down, relaxing)	1121/1496	81.6
Prescription pain medications (at least one of the drug classes below)	1314/1613	81.5
Analgesics (NSAIDs, Paracetamol, Aspirin, Metamizol)	883	56.0
Antidepressants	714	46.4
Muscle relaxants	298	18.5
Weak opioids	262	17.6
Strong opioids	119	8.4
Anticonvulsants (Pregabalin, gabapentin)	104	7.6
Self-management, physical modalities (Local and whole body warmth, thermal bathes))	1038/1549	67.0
Aerobic exercise (Aquatic exercise, walking/jogging, swimming, cycling)	902/1555	58.0
Physical therapies, manual (Chirotherapy, osteopathy, massage, lymph drainage, physiotherapy)	853/1619	52.7
CAM – medication (Homeopathy, dietary supplements, vitamins- and mineral nutrients, other CAM-drugs)	527/1497	35.2
CAM - diet (change of diet, elimination diet, fasting cure, vegetarian diet)	513/1484	34.6
Physical therapies, technical (Acupuncture, local injections, magnetic field, laser, TENS)	452/1584	28.5
Psychotherapy (Cognitive-behavioral therapies, psychodynamic therapies,other types of psychotherapy)	368/1517	24.2
Relaxation training (Autogenic training, progressive muscle relaxation)	357/1542	23.1
CAM - movement (Yoga, Tai Chi,Qi-Gong, dance- and music therapy)	278/1510	18,4
Psychological therapies (Biofeedback, hypnosis)	19/1423	1.3

* Note: The discrepancies between the number of persons in the different rows are due to missing items.

### Effectiveness and side effects

The highest average effectiveness was attributed to whole body warmth therapies (biosauna, infrared cabin, warmth bathes), thermal bathes, FMS education and resting and local warmth therapy (see Table 3). The highest average side effects were attributed to strong opioids, local cold therapy, gamma-amino-butyric acid analogues (GABA) (pregabalin, gabapentin), tramadol and opioid transdermal opioid systems (see Table 4).

**Table 3:** Top ten of most effective management strategies (0 = no benefit; 10 = maximum benefit)

**Effectiveness**			
**Order**	**Management strategy**	**N; Mean benefit (SD)** (0-10) *	**High benefit (8-10)** **N (%) **
1	Whole body warmth therapy	984; 7.1 (2.6)	507 (51.5)
2	Thermal bathes	699; 7.0 (2.7)	354 (50.6)
3	FMS education	649; 6.8 (2.7)	304 (46.8)
4	Resting	1296; 6.6 (2.5)	519 (40.1)
5	Local warmth therapy	1088; 6.6 (2.5)	452 (41.6)
6	Lymph drainage	481; 6.4 (2.8)	197 (41.0)
7	Functional training **	781; 6.2 (2.7)	271 (34.7)
8	Warm bathes	681; 6.1 (2.9)	250 (36.7)
9	Osteopathy	445; 6.0 (3.1)	177 (38.8)
10	Dance therapy	159; 6.0 (2.9)	60 (37.7)

* Note: The discrepancies between the number of persons in the different rows are due to missing items.

** Combination of land- and water-based exercises (stretching, aerobic exercise) guided by a licensed trainer; the costs are covered for two years by German health insurance companies if prescribed by a physician.

The efficacy ratings of all management strategies are available on request.

**Table 4:** Top ten of most harmful (side effects) management strategies (0 = no harm; 10 = maximum harm)

**Side effects**			
**Order**	**Management strategy**	N; Mean harm (0-10)(SD) *	**High harm (8-10)** N (%) *
1	Strong opioids	133; 5.4 (3.6)	49 (36.8)
2	Local cold therapy	367; 5.1 (4.1)	148 (40.3)
3	Anticonvulsants (Pregabalin, Gabapentin)	198; 5.0 (3.7)	69 (34.9)
4	Tramadol	365; 4.8 (3.6)	110 (30.1)
5	Transdermal opioids	115; 4.7 (3.8)	35 (30.4)
6	Duloxetine	192; 4.5 (3.7)	56 (29.2)
7	Amitriptyline	598; 4.5 (3.5)	157 (26.3)
8	Tramadol combined with paracetamol	82; 4.3(3.7)	20 (24.4)
9	Tilidine	262; 4.3 (3.5)	65 (24.8)
10	Whole body cold therapy	244; 4.1 (4.2)	79 (32.4)

* Note: The discrepancies between the number of persons in the different rows are due to missing items.

The ratings of side effects of all management strategies are available on request.

The frequency of use and the perceived benefits and harms of some therapies depended on some demographic (e.g. member of self-help group) and some clinical characteristics (e.g. comorbid inflammatory rheumatic disease and probable depressive disorder) (Jung et al., submitted).

## Discussion

### Summary of main results

We conducted the first European FMS consumer reports. 1661 FMS patients participated. In contrast to randomised controlled trials, patients with comorbid inflammatory rheumatic diseases and mental disorders were included. Self-management strategies were the most frequently types of interventions currently used by the participants. Participants attributed the highest effectiveness to the relief of FMS-symptoms by non-pharmacological treatments (warmth therapy, balneotherapy, education). In contrast, strong opioids and GABA-analogues (pregabalin, gabapentin) were associated with the strongest side effects.

### Comparison with other studies

The most frequently used therapies of the German and of the NFA-study [15] were resting and distraction. Aerobic exercise, prescribed analgesics and strength training were more frequently used by German compared to US survey participants. Antidepressants, prescribed sleep medication, nutritional supplements, massage and cold therapy were more frequently used by US than by German consumers. Both US and German survey participants indicated that resting, heat modalities and massage were the most effective therapies. German FMS-consumers reported less benefits from drug therapies than US American FMS-consumers (see Table 5).

**Table 5:** **Comparison of selected management strategies currently used by US- (members of the National Fibromyalgia Association) (N = 2596) [14] and German FMS-consumers (N = 1661) (in descending order of frequency according to US-survey)**[14]**(0 = no effectiveness or harm; 10 = maximum effectiveness or harm)**

**Management strategy**	**US consumers** **%; Mean effectiveness (0-10) (SD)**	**German consumers** % *; Mean effectiveness (0-10) (SD)
Resting	86; 6.3 (2.5)	82; 6.6 (2.5)
Distraction	80; 4.7 (2.5)	86; 5.8 (2.3)
Heat modalities	74; 6.3 (2.3)	54; 6.6 (2.5)
Nutritional supplements	68; 3.8 (2.8)	20; 4.0 (3.0)
Prescription pain medication	66; 6.3 (2.4)	82; 4.8 (2.3)
Gentle walking	64; 4.6 (2.6)	80; 5.9 (2.9)
Prescription antidepressants	63; 6.2 (2.8)	46; 4.1 (3.1)
Stretching	62; 5.4 (2.6)	44; 5.8 (2.6)
Prescription sleep medication	52; 6.5 (2.7)	7; 5.0 (3.0)
Relaxation	47; 5.1 (5.5)	49; 5.9 (2.7)
Massage	43; 6.1 (2.8)	25; 6.0 (2.8)
Aerobic exercise	32; 5.0 (3.0)	58; 5.4 (2.7)
Cold therapy	30; 4.8 (2.8)	10; 4.0 (3.5)
TENS	21; 4.3 (2.9)	17; 3.5 (2.8)
Strength training	18; 4.3 (2.9)	39; 5.3 (2.7)
Pain clinic	17; 4.8 (3.1)	4; 5.9 (3.3)
Acupuncture	15; 4.5 (3.5)	11; 4.4 (3.3)
Cognitive-behavioral therapy	8; 4.3 (3.2)	11; 5.5 (3.0)
Hypnosis	3; 2.5 (2.9)	1; 4.5 (3.3)

* Details are available on request.

Internet-based consumer reports can provide additional information on the effectiveness and side effects of therapies. “PatientsLikeme” is a social networking health internet webpage that enables its members to share conditions, treatment, and symptom information in order to monitor their health over time and learn from real-world outcomes. Patients can evaluate the effectiveness and type of side effects of drug therapies. 2084 (12%) of the FMS-patients registered in PatientsLikeme were currently on treatment with duloxetine and 1899 (11%) with pregabalin. The majority of the patients attributed a moderate efficacy to all of these drugs. 542 FMS-patients reported discontinuation of duloxetine and 687 to stopped pregabalin because of adverse effects [19].

### Limitations

Methodological problems such as retrospective design, lack of a control group, unspecified time frame, global assessment of effectiveness and side effects and lack of other assessment instruments than self-reports are inherent in effectiveness studies in a natural design such as used in the consumer reports [20].

Additional major methodology limitations of the design of this study were as follows: the different modalities of the distribution and recollection of the questionnaires led to a relevant number of missing data; the unknown response rate of the participants recruited by self-help organisations limited the generalizability of the results; and selection bias with respect to patients and study investigators preferring non-pharmacological and complementary therapies could not be excluded. While this study is the largest European consumer review to-date, the results still represent a relatively small sample of the overall available population.

## Conclusions

Consumer reports represent a complementary source of information on therapeutic effectiveness and side effects, and may support treatment decisions of patients and physicians.

The German FMS consumer reports highlight the importance of self-management strategies and non-pharmacological treatment options in the long-term management of FMS, and challenge the strong recommendations for drug therapy of FMS given by FMS-guidelines [11-13].

Health webpages should allow the evaluation of non-pharmacological therapies, and future FMS-consumer reports should include measures of function to provide a broader context for study findings.

## Competing interests

WH received honoraria for education lectures from Eli-Lilly, Pfizer and Janssen-Cilag and a congress travel grant by Eli-Lilly. TW and AW were investigators in a study of pregabalin, sponsored by Pfizer. FW received a grant for the National Data Bank for Rheumatic Diseases from Lilly Research Laboratories in 2009 to support the American College of Rheumatology 2010 critera study,. The other authors have no conflicts of interest to declare.

## Authors’ contributions

The study was designed by all authors. All authors except EJ and FW recruited patients for the study. EJ and WH extracted and analysed the data. All authors were involved in drafting the manuscript or revising it critically for important intellectual content and gave final approval of the version to be published.

## Authors’ information

WH is responsible for the coordination of the German interdisciplinary guideline on the management of fibromyalgia syndrome. MG, HKB, JH, TW and AW are members of the German FMS-guideline group. EJ is a post-doctorate in medicine. FW is the first author of the paper on ACR 2010 preliminary diagnostic criteria and their modification for survey research.

## Pre-publication history

The pre-publication history for this paper can be accessed here:

http://www.biomedcentral.com/1471-2474/13/74/prepub
